# Study of Niobium-Based Coatings and Silver Incorporation for Biomedical Applications

**DOI:** 10.1021/acsomega.5c13198

**Published:** 2026-03-31

**Authors:** Ana Elisa Dotta Maddalozzo, Júlia Abdala, Amanda Santi, Janete Eunice Zorzi, Lucia Vieira, Mariana Roesch-Ely, Cesar Aguzzoli

**Affiliations:** † Graduate Program in Materials Science and Engineering, University of Caxias do Sul, 95070-560 Caxias do Sul, Rio Grande do Sul, Brazil; ‡ 67655 Universidade do Vale do Paraíba, São José dos Campos, São Paulo 12244-000, Brazil; § Graduate Program in Biotechnology, University of Caxias do Sul, 95070-560 Caxias do Sul, Rio Grande do Sul, Brazil; ∥ Independent researcher, 95170-444 Farroupilha, Rio Grande do Sul, Brazil

## Abstract

Surface engineering has established itself as a fundamental strategy for enhancing the performance of materials in critical environments, particularly in the biomedical sector, where resistance to corrosion, wear, and microbial contamination is essential. In this context, the aim of this work was to develop and characterize niobium-based coatings incorporating silver, targeting applications in the biomedical field. NbN and Nb_2_O_5_ coatings were obtained by magnetron sputtering and subsequently modified through ion plating with silver ions. The results showed that the films exhibit promising physicochemical and biological properties. The coatings demonstrated excellent biocompatibility, corrosion and wear resistance, and bactericidal activity, being effective in preventing biofilm formation without presenting cytotoxicity. The research confirms the effectiveness of surface engineering as a sustainable and efficient approach to mitigating corrosion, wear, and microbial contamination in critical environments. Future studies may focus on long-term evaluations and industrial-scale feasibility.

## Graphical abstract

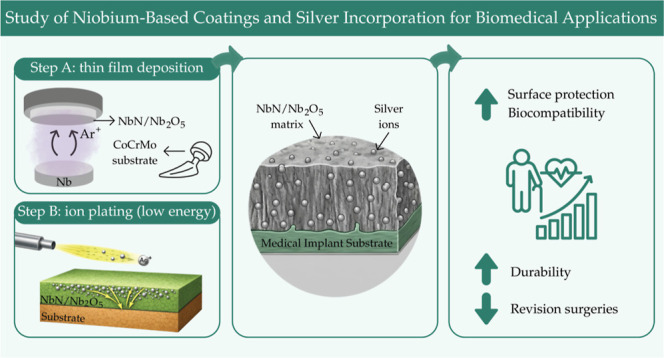

## Introduction

Surface engineering plays a pivotal role in enhancing the performance of materials, particularly with respect to tribological behavior, corrosion resistance, and biocompatibility.^1^ Among the wide range of available techniques, magnetron sputtering stands out as a versatile and reliable method for depositing high-quality thin films over relatively large areas, ensuring excellent adhesion to the substrate. Operating under vacuum, this process significantly reduces contamination in the resulting coatings and is regarded as more environmentally sustainable compared to conventional deposition techniques.^2^

Given these advantages, the present study focuses on the development of niobium-based thin films deposited via magnetron sputtering onto metallic substrates, aiming to address critical challenges in the biomedical sector. Coatings such as niobium nitride (NbN) and niobium pentoxide (Nb_2_O_5_) were selected due to their wide range of desirable properties, including biocompatibility, corrosion resistance, wear resistance, and fatigue strengt, characteristics that make them promising candidates for applications requiring long-term durability and chemical stability.^3,4^

To further enhance the functional performance of these films, silver was incorporated into the surfaces via an ion plating technique, improving antibactericial activity and inhibiting biofilm formation.^5^ The implantation process was adapted by the research group to achieve a higher ionization degree of the metallic ions while employing low bias energies (<5 keV), overcoming limitations typically associated with conventional ion implantation.

The choice of substrate was guided by both technical criteria and practical application relevance. CoCrMo alloy was selected owing to its widespread use in orthopedic and dental prostheses, driven by its outstanding biocompatibility, corrosion resistance in physiological environments, and superior mechanical properties such as high hardness and wear resistance. This alloy is primarily composed of cobalt, chromium, and molybdenum, elements that ensure microstructural stability and promote the formation of a protective passive layer.^6^

This strategic selection of materials and deposition methods enabled a comprehensive evaluation of the coatings’ performance under demanding conditions. A series of physicochemical, structural, mechanical, and biological characterizations were carried out, including thickness measurements, crystal structure analysis, chemical composition assessment, wear and corrosion resistance testing, as well as cytotoxicity and biofilm formation assays. These analyses validated the effectiveness of the proposed approach and confirmed the feasibility of its practical application.

In light of the growing demand for advanced materials capable of operating in extreme environments, this work addresses the pressing need for functional solutions that combine mechanical strength, corrosion resistance, and antimicrobial properties. The primary motivation lies in overcoming persistent challenges in the biomedical field, particularly those associated with implant-related infections and corrosion-induced failures.

While the use of niobium-based coatings and silver nanoparticles has been individually explored, there remains a clear gap in the literature regarding their synergistic application in critical scenarios such as those targeted in this study, and this research proposes an innovative surface engineering strategy capable of bridging this gap and delivering sustainable, high-performance technological solutions with direct implications for public health. Compared with recent literature on silver-based antimicrobial coatings for biomedical applications,^7,8^ the present work distinguishes itself by employing a low-energy ion plating approach to implant silver ions directly into Nb_2_O_5_ and NbN thin films rather than relying on traditional nanoparticle deposition or high-energy implantation. In this research, Ag^+^ ions are implanted at significantly lower implantation energies, resulting in a near-surface distribution of antimicrobial species that enhances ion availability within the critical first 24 h after implantation. Moreover, the approach applied to Nb_2_O_5_ and NbN specifically integrates ionic silver species into ceramic-like coatings not typically explored in the referenced reviews, which focus predominantly on titanium or polymeric substrates, thus expanding the applicability of silver-based antimicrobial strategies to transition metal nitride and oxide thin films for implants. This unique combination of material systems and low-energy implantation enhances antibacterial functionality with controlled ion release and improved interface stability, offering a distinctive path forward beyond classical AgNP coatings discussed in the current literature.

## Methodology

### Sample Preparation

The CoCrMo substrates were sanded using abrasive papers with grit sizes of 400, 600, 800, and 1000, followed by an ultrasonic bath in P.A. acetone for 30 min.

### Deposition of NbN

The deposition of niobium nitride (NbN) was carried out on the surface of CoCrMo and silicon samples (the latter used to improve film characterization in specific analyses). The substrates were placed inside the vacuum chamber of the magnetron sputtering system, which was evacuated to a base pressure of 3 × 10^–7^ mbar (high vacuum). Subsequently, by applying an RF power source, high-purity argon gas (LINDE −99.9999% purity) was ionized to generate plasma. The resulting positively charged Ar cations were accelerated toward the niobium target (99.9% purity), which was held at a negative potential and positioned 5 cm from the sample. Due to linear momentum transfer during this collision, atoms from the target were ejected.

In addition to argon, high-purity nitrogen gas (AGA −99.9999% purity) was also introduced during deposition, thus characterizing the process as reactive magnetron sputtering. In this way, Nb atoms ejected from the target reacted with nitrogen ions, forming the ceramic compound NbN. The deposition parameters are listed in Table 1.

**Table 1:** Deposition Parameters for NbN Films^a^

base pressure	work pressure	argon flow	nitrogen flow	rf power	temperature	deposition time
3 × 10^–7^ mbar	8 × 10^–3^ mbar	13 sccm	2.6 sccm	100 W	300 °C	three distinct durations: 15 min; 1 h; 2 h

a Parameters selected based on literature.^3^

Before deposition, the samples underwent na in situ etching step to clean the surface and enhance film–substrate adhesion. This procedure was also performed inside the magnetron sputtering vacuum chamber, at the same base pressure used for the NbN deposition. In this case, however, the argon plasma was ignited at the sample holder instead of the target, allowing Ar ions to remove surface contaminants such as oxides through physical sputtering. For this purpose, 50 sccm of Ar, an RF power of 50 W, and a process time of 10 min were employed.

### Fabrication of the Nb_2_O_5_ Target

The niobium oxide target was produced from optical-grade Nb_2_O_5_ powder supplied by Brazilian Metallurgy and Mining Company (CBMM). The specifications provided by the company are shown in Table 2.

**Table 2:** Specifications Provided by CBMM for the Nb_2_O_5_ Powder

crystalline phases at room temperature	purity	particle size	phase transformation upon heating
orthorhombic and monoclinic	99.9%, with tantalum impurity (<1500 ppm)	D50–40 μm and D90–70 μm	H–monoclinic >900 °C

For target production, 22 g of powder were pressed and shaped into a disk measuring 5.1 cm in diameter and 0.4 cm in thickness. To ensure particle cohesion, 10 wt % poly­(vinyl alcohol) (PVA) in an aqueous solution was used as a binder. Several trials were conducted until the optimal sintering parameters were determined. In the final procedure, the target was first presintered at 1100 °C in a Sanchis BTT furnace for 4 h, followed by sintering at 1375 °C in a Sanchis furnace for an additional 4 h. Cooling occurred gradually as the furnace cooled down naturally after the process ended. Sintering was performed in two stages due to dimensional constraints: initially, the disk would not fit into the furnace capable of reaching 1375 °C. After shrinkage from presintering at 1100 °C, the target fit into the higher-temperature furnace. The final density of the target was 2.7 g/cm^3^.

To investigate the phase transformation induced by sintering and verify the information provided by the supplier, X-ray diffraction (XRD) analyses were performed on the powder at room temperature and after sintering at 900 °C, 1000 °C, and 1100 °C. The results are shown in Figure 1.

**Figure 1:**
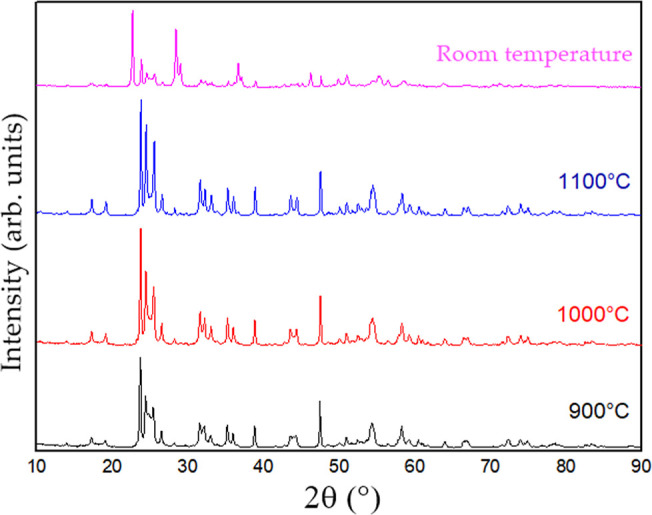
X-ray diffractograms of Nb_2_O_5_ powder at different temperatures.

The diffractogram of the as-received powder was similar to that of the sintered samples, with some peaks at room temperature exhibiting lower intensity. A shift toward lower diffraction angles was observed, indicating stress formation during heating. This stress may explain the shrinkage of the sample during sintering.

Comparison with diffractograms reported in the literature^9–11^ confirmed that the crystalline structure of niobium oxide is monoclinic, in agreement with the specifications provided by CBMM. Notably, one of the cited studies also used the Nb_2_O_5_ powder supplied by CBMM.^12^

### Deposition of Nb_2_O_5_

Niobium oxide was deposited onto CoCrMo and silicon samples. During deposition, the base pressure was 3 × 10^–7^ mbar, with power and time parameters varying as shown in Table 3. An argon gas flow (LINDE −99.9999% purity) of 9 sccm was used, resulting in a working pressure of 5 × 10^–3^ mbar. The process was carried out at room temperature.

**Table 3:** Deposition Parameters for Nb_2_O_5_ Films^a^

Nb_2_O_5_ Sample	sample name	deposition time	RF Power
1	Nb_2_O_5_-30 min-70W	30 min	70 W
2	Nb_2_O_5_-30 min-90W	30 min	90 W

a Parameters selected based on literature.^13,14^

Prior to deposition, the samples underwent an in situ etching process using 50 sccm of Ar, an RF power of 50 W, and a process time of 10 min.

### Silver Implantation

Silver ion implantation was carried out using the ion plating technique. The base pressure was 3 × 10^–7^ mbar, and the process parameters are presented in Table 4.

**Table 4:** Parameters for Silver Implantation^a^

	coil current (A)	filament current (A)	electron emission current (mA)	BIAS (keV)	thickness gauge voltage (V)
**sample 1** (**Ag** – **5 keV** – **3 V**)	–0.23	16.2	25	–5	3
**sample 2** (**Ag** – **10 keV** – **3 V**)	–0.25	15.4	25	–10	3
**sample 3** (**Ag** – **5 keV** – **6 V**)	–0.25	16.7	102	–5	6
**sample 4** (**Ag** – **10 keV** – **6 V**)	–0.30	16.6	92	–10	6

a Parameters selected based on previous studies conducted by the research group.

### Analysis of Thin Film Thickness and Quantification of Implanted Silver

The thickness of the thin films was analyzed by X-ray fluorescence (XRF) using a Shimadzu EDX-6000 system. The results of this analysis are expressed in μg/cm^2^. Therefore, to convert the obtained values to nanometers, eq 1 was applied

$$\frac{Result\;in\;\mu g/{cm}^{2}}{Theoretical\;film\;density\left(g/{cm}^{3}\right)\times 10^{6}}\times 10^{7}=Result\;in\;nm$$

The theoretical densities used for the calculations were 4.6 g/cm^3^ for niobium oxide and 8.5 g/cm^3^ for niobium nitride. Furthermore, the XRF analysis also enabled the quantification of silver implanted in the samples.

### Analysis of Coating Stoichiometry

The stoichiometry of the films was analyzed using a simulation with the free software DTSA II, developed by the National Institute of Standards and Technology (NIST). This program takes into account data obtained from energy-dispersive X-ray spectroscopy (EDS), which in this study was performed using an X-act detector from Oxford Instruments, United Kingdom. Furthermore, the stoichiometry calculation is based on the film thickness and density. The value used for this parameter was the one found in the literature, as reported on the previous section. The measurements were performed at an accelerating voltage of 15 kV and a working distance of 15 mm.

### Grazing Incidence X-Ray Diffraction (XRD)

Grazing incidence X-ray diffraction was performed to analyze the crystalline structure of the films, using an X-ray diffractometer (Model XRD-6000, Shimadzu, Japan) with Cu Kα radiation of λ = 1.5406 Å. A grazing incidence angle of 1° was employed, while the 2θ angle was varied between 10° and 100°.

### Microabrasive Wear Test by Rotating Ball

The wear test was conducted using a Calotest device (CSM, Centre Suisse d’Electronique et de Microtechnique) based on the microabrasive wear method by rotating ball.^15^ In this technique, a diamond abrasive suspension with a particle diameter of 0.5 μm was dripped onto a steel ball by means of a peristaltic pump. A magnetic stirrer was also used to maintain the suspension in constant motion. The contact force of the ball on the sample surface was measured with a load cell. The sliding of the ball against the sample surface generates craters whose diameter related to the wear resistance of the sample and test conditions were measured using an optical microscope and image acquisition software. The wear coefficient is obtained as the slope of the line fitted to the wear volume versus sliding distance data. Five measurements were taken per sample, in different regions, and the analysis time ranged from 60 to 210 s with intervals of 30 s. The wear coefficient, κ, can be calculated using eq 2

$$\kappa =\frac{\pi b^{4}}{32LdF_{N}}$$

where *L* is the sliding distance traveled by the ball on the sample, *F*_N_ is the normal load on the sample, *b* is the diameter of the formed crater, and *d* is the diameter of the ball (⌀ 25.4 mm). The analysis was performed on pure CoCrMo samples and coated samples.

### Monte Carlo Simulation

To obtain simulations of the Ag^+^ ion depth profiles, the Monte Carlo method was employed using the CASINO software developed at the University of Cambridge. Simulation parameters were adjusted according to the ion characteristics (silver), the target implantation materials (NbN and Nb_2_O_5_), and the BIAS voltages applied during the process (5 and 10 keV).

### Cell Viability Test

DMEM culture medium supplemented with 10% fetal bovine serum (FBS) and 1% penicillin/streptomycin (P/S) was placed in contact with all samples for 24 h at 37 °C in a 5% CO_2_ atmosphere. Subsequently, cytotoxicity was assessed using the MTT assay, following ISO 10993-12 protocols. This technique is based on the reduction of MTT (3-(4,5-dimethylthiazol-2-yl)-2,5-diphenyltetrazolium bromide) by mitochondrial dehydrogenase enzymes forming formazan crystals. MG63 cells were seeded at a density of 5 × 10^4^ cells/mL in 100 μL of DMEM supplemented with 10% FBS and 1% P/S. Upon reaching 70%–80% confluence, cells were treated with extraction solutions obtained by immersing the samples in culture medium for 1 and 2 days. Plates were incubated at 37 °C in 5% CO_2_. The medium was removed, and 1 mg/mL of MTT in serum- and antibiotic-free medium was added to the wells. Plates were incubated at 37 °C for 2 h in a humidified atmosphere with 5% CO_2_. Afterward, the MTT solution was removed, and formazan crystals were dissolved in 100 μL of DMSO. Spectrophotometric readings were taken at 570 nm using a microplate reader (Me2 Spectra, Molecular Devices, USA), and results were expressed as percentage viability. The absorbance of the control sample containing only the CoCrMo substrate represented 100% viability, and treated cell values were calculated as a percentage thereof. This assay was performed in duplicate using samples coated with NbN and Nb_2_O_5_ films implanted with Ag^+^. The results represent the average of both coatings in each of the two replicates.

### Evaluation of Antibacterial Activity

For this analysis, the bacterial strains *Staphylococcus aureus* and *Escherichia coli* were used. The inocula were diluted in sterile saline solution to match the turbidity of the 0.5 McFarland standard, resulting in an approximate cell density of 1–2 × 10^8^ CFU/mL. Subsequently, plates were inoculated using a sterile swab. In the diffusion test, Mueller–Hinton agar plates were uniformly inoculated with the microorganisms. The samples were then placed on the inoculated agar plates. The plates were incubated for 24 h at 37 ± 1 °C, and inhibition zones were observed. Additionally, DAPI (4′,6-diamidino-2-phenylindole), a fluorescent dye emitting bright blue fluorescence used to stain DNA in live cells, was employed. After 24 h of incubation, samples were removed and 10 μL of DAPI dye was added to the area that was directly in contact with the microorganisms. Samples were then visualized under a fluorescence microscope, where the presence of bright blue fluorescence indicated the presence of bacteria. A representative schematic of the analysis is shown in Figure 2, created with the aid of Biorender software.

**Figure 2:**
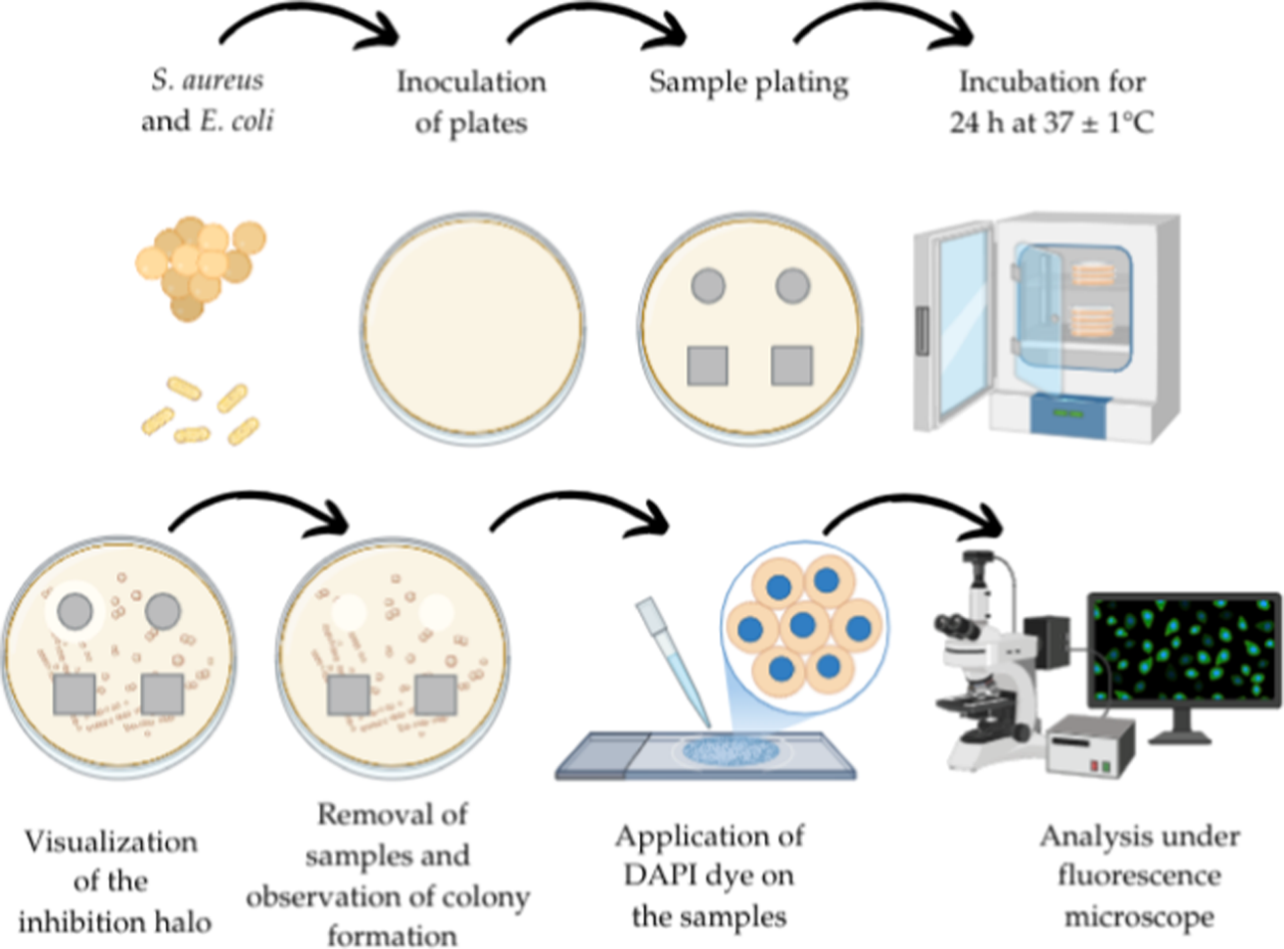
Antimicrobial activity of the samples. The image was created with the aid of Biorender software (free domain).

### Evaluation of Wettability and Surface Roughness

The sessile drop technique was employed to measure the contact angle of the films, using a goniometer (Model 300, SEO Phoenix, South Korea). To determine the wettability of the films, three droplets per sample were analyzed, with each droplet measured ten times. Distilled water was used as the test liquid. Samples were stored at room temperature and protected from light exposure. The average surface roughness (Ra) of the samples was assessed using a profilometer (Model 112, Taylor Hobson, UK), performing measurements along three 10 mm tracks in three different directions (0°, 45°, and 90°).

### Quantitative Corrosion Testing

Open Circuit Potential (OCP) and tribocorrosion tests were performed using a Bruker CETR-UMT & CETR-APEX tribometer (version 1.138.259+). Three distinct electrodes were employed: a reference electrode, a counter electrode, and a working electrode. The reference electrode used was a silver chloride electrode (Ag/AgCl, Chenhua Chi111), immersed in a 1 mol potassium chloride (KCl) solution prepared by dissolving 7.45 g of KCl in 100 mL of bidistilled water. This type of electrode has a stable and well-known potential, which is essential for isolating the electrochemical reaction at the working electrode by maintaining constant redox species concentrations. The counter electrode was a platinum wire (CHI 115, 0.5 × 35 mm), facilitating charge flow to complete the electrochemical circuit, essential for electron transfer analyses. The working electrode was represented by the sample under test.

Sample surface preparation involved polishing the untreated area with 1200-grit sandpaper, followed by cleaning with 70% ethanol and complete drying. Sterile fetal bovine serum (FBS, Vitrocell Embriolife) preheated to 37 °C in a water bath was used to simulate body environment.

Samples were mounted in a tribocorrosion cell, positioned in the lower groove of the container and secured by four screws to hold them flat and firmly in the sample holder. After installation, approximately 100 mL of FBS was added to immerse the sample, ensuring electrical contact with the reference and counter electrodes. Electrical continuity was verified using a multimeter.

With the cell assembled, the Open Circuit Potential was measured before the tribocorrosion test to assess the ionic exchange stability between the solution and electrodes, without applying load or potential to the sample. The stabilization time was 1 h (3600 s). OCP stabilization was considered achieved when potential variation did not exceed 2 mV within a 10 min interval, a criterion observed throughout different spectrum sections. In interpreting OCP results, more negative potentials relative to the reference electrode indicate higher oxidation tendencies and lower corrosion resistance, while more positive potentials reflect nobler behavior, indicating greater stability and reduced corrosion susceptibility.

Following OCP stabilization, the tribocorrosion test was performed with the assembly unchanged. A 100 N load cell was used, applying a constant normal force of 5 N at a speed of 1 mm/s. The total test duration was 3 h. Tribocorrosion results from the synergistic interaction between mechanical stresses and chemical action in corrosive environments, causing wear due to simultaneous tribological and electrochemical effects.

### X-Ray Photoelectron Spectroscopy (XPS)

The chemical composition and bonding states of elements present on the sample surfaces were determined by X-ray photoelectron spectroscopy (XPS) using an Omicron Multiprobe Sphere instrument with Al Kα radiation (1253.6 eV) at a takeoff angle of 60° and an energy resolution of 0.9 eV.

Figure 3 provides a summarized overview of the research project stages.

**Figure 3:**
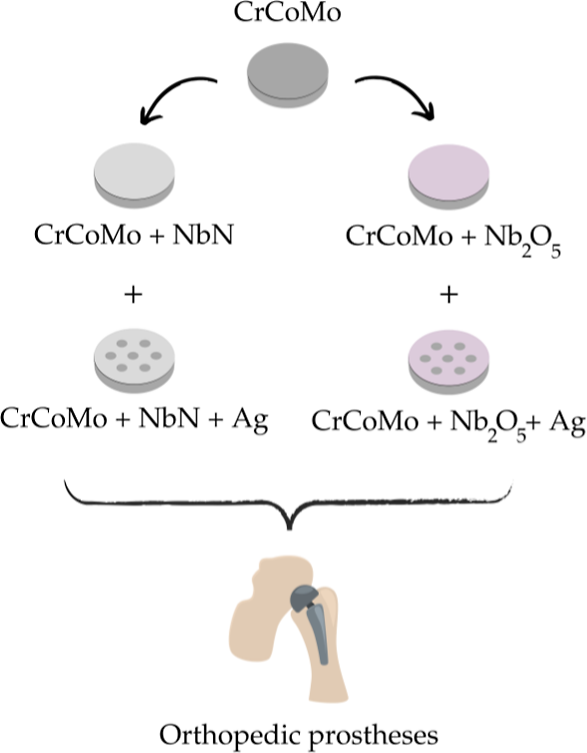
Schematic overview of the project stages. The image was created with the aid of Canva (free domain).

## Results and Discussion

The following sections present the characterization procedures and results obtained for the deposited coatings. The overall objective was to identify the optimal deposition condition for each coating—one for niobium nitride and one for niobium oxide—based on a sequential and rational evaluation workflow. The selection proceeded as follows: initially, one niobium nitride coating and one niobium pentoxide coating were chosen according to their performance in the microabrasive wear test, specifically privileging the films exhibiting the lowest wear coefficient. These selected coatings then underwent silver ion implantation via ion plating and were subsequently evaluated through the agar diffusion antibacterial assay (halo inhibition test). The coatings showing the most promising antibacterial performance were further subjected to electrochemical corrosion testing. From these results, one optimal coating condition was identified for NbN and another for Nb_2_O_5_. Throughout this process, complementary physicochemical and biological characterizations were conducted to support and refine the interpretation of the functional performance of the coatings.

### Analysis of Thin Film Thickness

The thickness of the NbN and Nb_2_O_5_ films was calculated using the X-ray fluorescence (XRF) technique, and the results are shown in Table 5.

**Table 5:** Thickness Measurements of NbN and Nb_2_O_5_ Films Obtained XRF Analysis

NbN samples	sample name	thin film thickness
1	NbN-15 min	35 nm
2	NbN-1 h	143 nm
3	NbN-2 h	281 nm

**Nb**_**2**_ **O**_**5**_ **samples**	**sample name**	**thin film thickness**
1	Nb_2_O_5_-30 min-70 W	113 nm
2	Nb_2_O_5_-30 min-90 W	159 nm

Thickness measurements of NbN samples 1, 2, and 3 show an increase with deposition time. Similarly, the Nb_2_O_5_ film deposited at higher RF power (90 W) exhibited greater thickness due to increased plasma density and enhanced argon ionization, which promote higher sputtering rates from the ceramic target.

The thickness values found for the NbN and Nb_2_O_5_ samples are consistent with literature reports, which range from 180 to 500 nm and exhibit desirable properties for orthopedic prosthesis applications, such as wear resistance, corrosion resistance, hardness, improved biocompatibility, and a surface characteristic change from hydrophobic to hydrophilic.^16–18^

### Analysis of Coating Stoichiometry

The stoichiometry values obtained through DTSA II simulation are listed in Table 6. It can be observed that the stoichiometries of the niobium oxide samples were nearly identical regardless of the deposition power used. Moreover, the measured values are very close to the theoretical stoichiometry, likely because the target was composed of Nb_2_O_5_ powder. Therefore, when deposition is not performed via reactive sputtering, the likelihood of achieving the desired stoichiometry is higher.^19^

**Table 6:** Stoichiometry of Niobium-Based Thin Films

NbN samples	sample name	stoichiometry
1	NbN-15 min	it was not possible to stipulate
2	NbN-1 h	Nb_2_N
3	NbN-2 h	NbN

**Nb**_**2**_ **O**_**5**_ **samples**	**sample name**	**stoichiometry**
1	Nb_2_O_5_-30 min-70 W	Nb_1,5_O_5_
2	Nb_2_O_5_-30 min-90 W	Nb_1,5_O_4,5_

In contrast, forming a compound by reaction between a gas and a solid target, as in the case of NbN, is influenced by many factors. Coating growth is a complex process governed by thermodynamics, kinetic energy, and intrinsic technique parameters such as temperature, gas pressure, and deposition time.^20,21^

The NbN sample deposited for 15 min could not be simulated, likely due to its very thin film thickness (33 nm). However, the 1 h coated sample exhibited Nb_2_N stoichiometry, while the 2 h sample showed NbN stoichiometry. The possible explanation for this difference is discussed in the next section.

Notably, Nb_2_N formation under conditions similar to those described here has been reported in the literature. Qi et al. produced Nb_2_N films with 1 h deposition at 300 °C, showing 65.4 at. % Nb and 33.3 at. % nitrogen, values close to those found in the DTSA II simulation.^22^

Energy-dispersive X-ray spectroscopy combined with quantitative simulation software such as DTSA II is known to present limitations for stoichiometry determination in very thin films, particularly due to substrate contributions and reduced X-ray generation volume. However, literature has shown that, under appropriate conditions, quantitative EDS can still yield reliable compositional information for nanoscale coatings. For example, de Oblitas et al.^23^ evaluated sub-100 nm metallic alloy films using EDS and validated the elemental quantification by direct comparison with Rutherford Backscattering Spectrometry (RBS), reporting good consistency between the two techniques. This demonstrates that, although EDS/DTSA II is not universally optimal and must be applied with caution, it remains a viable method for thin-film stoichiometry when its constraints are recognized and results are interpreted within their metrological context.

### Grazing Incidence X-Ray Diffraction (XRD)

The X-ray diffraction (XRD) pattern obtained for the CoCrMo substrate is shown in Figure 4, whereas those corresponding to niobium oxide and niobium nitride are presented in Figures 5 and 6, respectively.

**Figure 4:**
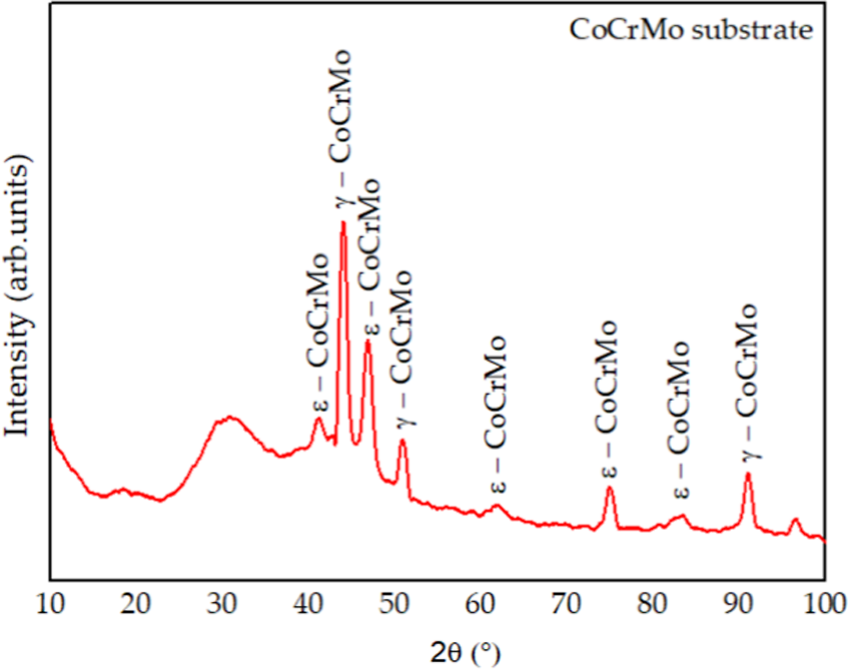
XRD pattern obtained for the CoCrMo substrate.

**Figure 5:**
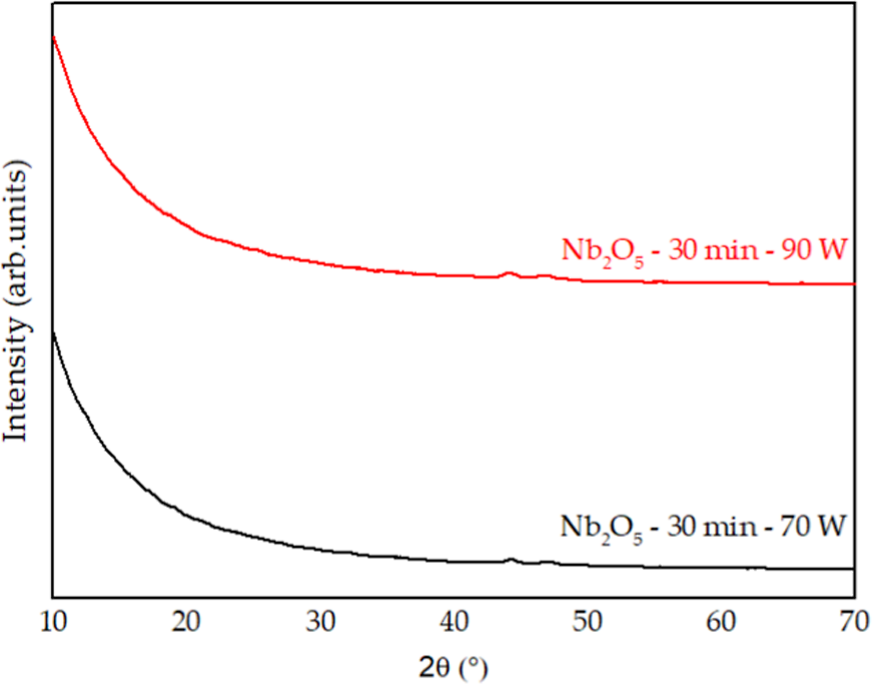
XRD patterns obtained for niobium oxide.

**Figure 6:**
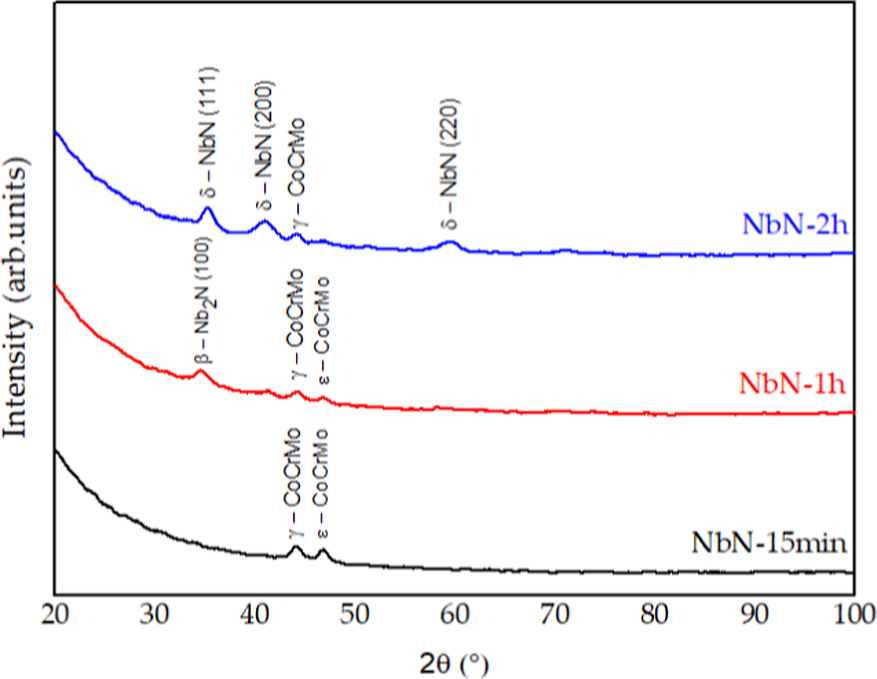
XRD patterns obtained for niobium nitride.

It can be observed that the niobium oxide films exhibit an amorphous structure. This result was expected, since several studies in the literature report the formation of amorphous niobium oxide when no substrate heating is applied.^24^ Ramírez et al. deposited amorphous Nb_2_O_5_ onto stainless steel with the aim of developing coatings for dental implant applications. Their results indicated that the coating enhanced cell adhesion, viability, and proliferation, thereby improving the overall performance of the implants. This improvement was associated with the increased hardness, enhanced corrosion resistance, and superior biological response provided by the material.^24^

The amorphous nature of the coatings can be advantageously exploited to enhance the corrosion resistance of metallic materials. It has been reported that amorphous films exhibit higher corrosion resistance compared to their crystalline counterparts, due to the absence of defects such as grain boundaries and dislocations, as well as their chemical homogeneity.^25^

The niobium nitride thin films also exhibited a predominantly amorphous character. At first, the thinnest film, produced with a 15 min deposition time, presented two peaks corresponding to the CoCrMo substrate, namely the face-centered cubic (γ) and the hexagonal close-packed (ε) phases. The film obtained after 1 h of deposition showed a mainly amorphous profile, with a low-intensity peak attributed to the hexagonal β-Nb_2_N phase.^26^ Finally, the coating deposited for 2 h, being the thickest, exhibited a greater number of peaks associated with the cubic δ-NbN phase.^27–29^ It is worth noting that the peak corresponding to the β–Nb_2_N (100) phase appears at the same 2θ position (35°) as the δ–NbN (111) phase. The distinction between the two can be made through the stoichiometric analysis discussed in the previous section, which confirmed that the film deposited for 1 h corresponds to Nb_2_N, whereas the 2 h film corresponds to NbN.

A possible explanation for this change in crystalline phase has been reported in the literature,^30^ suggesting that the phase of NbN films depends on the nitrogen pressure. At lower pressures, the β–Nb_2_N phase is observed, while at higher pressures, the δ–NbN phase becomes predominant. In the present work, the nitrogen pressure was kept constant; however, a similar effect may be attributed to increased nitrogen diffusion over longer deposition times. This could explain the observed phase transitions in the samples. Additional evidence supporting this interpretation lies in the known sequence of nitride formation reported in the literature as the nitrogen content increases: α–NbN → β–Nb_2_N → γ–Nb_4_N_3_ → δ–NbN → Nb_4_N_5_.^31^ It is also worth emphasizing that the formation of amorphous niobium nitride is not unusual in the current state of the art, as the compound has been reported to form at 300 °C,^32^ consistent with the conditions used in this study.

### Microabrasive Wear Test by Rotating Ball

The wear coefficient results are presented in Table 7, showing that the values obtained for the coated samples were lower than that of the uncoated CoCrMo substrate (0.91 × 10^–12^ m^2^/N). This indicates a reduction in wear volume for the samples coated with ceramic thin films, as these films exhibit higher hardness than the metallic substrate. This finding is consistent with literature reports demonstrating that thin films of NbN and Nb_2_O_5_ enhance wear resistance compared to metallic substrates commonly used in biomedical applications, such as Ti6Al4 V,^33^ 316L stainless steel,^34,35^ and CoCrMo alloys.^36^

**Table 7:** Measured Wear Coefficients for the Tested Samples

sample	wear coefficient κ (10^–12^ m^2^/N)
CoCrMo	0.91
Nb_2_O_5_-30 min-70 W	0.45
Nb_2_O_5_-30 min-90 W	0.42
NbN-15 min	0.54
Nb_2_N-1 h	0.37
NbN-2 h	0.43

The inverse relationship between hardness and wear volume is described by Archard’s wear equation, where Q is the wear rate, ξ is a dimensionless constant representing wear severity, H is the surface hardness of the material, and N is the normal load at the contact interface between the ball and the sample

$$Q=\xi \frac{N}{H}$$

When analyzing similar thin films, for instance Nb_2_O_5_, an inverse relationship is observed between coating thickness and wear coefficient. This trend is consistent with the literature, which predicts that wear rate decreases as the film thickness increases.^37^ The Nb_2_O_5_ coating deposited at 90 W exhibited a thickness of 159 nm, whereas that deposited at 70 W had a thickness of 113 nm. Consequently, the former presented a lower wear coefficient due to its greater thickness, resulting from the higher deposition power. However, since the difference in thickness between the two coatings is not substantial, the variation in wear volume is also relatively small.

For the NbN coatings, a similar trend was observed. The film deposited for 15 min, being the thinnest (35 nm), displayed the highest wear coefficient. However, this trend was reversed for the films deposited for 1 and 2 h, with thicknesses of 143 and 281 nm, respectively. Although the 2 h film was thicker, its wear coefficient was higher than that of the 1 h film. This occurs because the 1 h film corresponds to Nb_2_N, whereas the 2 h film is stoichiometric NbN. Literature reports that β–Nb_2_N films, such as the one identified in this study, exhibit higher hardness than δ–NbN films due to the higher atomic packing density of the hexagonal close-packed structure compared to the cubic structure.^38^

The results obtained in this work are particularly relevant for implant applications, where components must move smoothly and efficiently to ensure proper body motion while minimizing wear rates and the risk of osteolysis.^39^ Moreover, an extended implant lifespan can be achieved, reducing the need for replacements and invasive surgical procedures.^40^

It is important to note that this tribological study presents certain limitations, such as the relatively low number of wear cycles, specific motion types, and the use of a diamond abrasive. Future studies simulating in vivo conditions—with millions of cycles using knee or hip joint simulators and physiological lubricants such as proteins, serum, or saline solutions—are necessary to better replicate the actual performance of the coatings.^41^

Based on the wear test results, the subsequent stages of this research focused on the best-performing coatings. Therefore, the niobium oxide thin film selected was that deposited at 90 W for 30 min, while the chosen niobium nitride film was that deposited for 1 h.

### Monte Carlo Simulation

Using the optimized parameters for the NbN and Nb_2_O_5_ films, a Monte Carlo simulation was performed to evaluate the penetration depth of silver ions into the coatings. The saturation depth of the ions is presented in Table 8. As expected, the penetration depth of silver ions increases with increasing implantation voltage, showing an average increase of approximately 47% across all cases analyzed.

**Table 8:** Saturation Depth for Silver Ions

sample	saturation depth (nm)
**Nb**_**2**_ **O**_**5**_ – **5 keV**	5.1
**Nb**_**2**_ **O**_**5**_ – **10 keV**	7.6
**Nb**_**2**_ **N** – **5 keV**	3.1
**Nb**_**2**_ **N** – **10 keV**	4.5

The penetration depth is inversely proportional to the density of the films. The theoretical density of Nb_2_N is 8.1 g/cm^3^, whereas that of Nb_2_O_5_ is 4.6 g/cm^3^. These values confirm the relationship between density and penetration depth, as the depth is greater for the less dense Nb_2_O_5_ film and lower for the denser Nb_2_N film. In addition to density, ion implantation also depends on the crystalline structure and the orientation of atomic planes of the base material.^42^ However, since the coatings in this study are amorphous, density is the primary factor influencing the observed results. The saturation depth is related to the energy loss of the ions, which occurs due to successive collisions with the atoms of the material.^43^

Unlike high-energy processes, low-energy ion implantation tends to concentrate ions near the surface, which facilitates their leaching and enhances their applicability as antibacterial agents. The literature shows that Ag^+^ ions implanted at depths similar to those in this study can effectively prevent bacterial adhesion. For example, Echeverrigaray et al.^44^ implanted silver into stainless steel at a saturation depth of approximately 2.5 nm using a 4 keV voltage. In this case, adhesion of *S. aureus* and *E. coli* was reduced by roughly 70%. In another study, Soares et al.^45^ implanted silver ions into titanium at 4 keV, achieving a penetration depth of around 4 nm. Diffusion tests on agar indicated that the samples exhibited bactericidal activity against *E. coli*, as no bacterial colony growth was observed.

### Quantification of Implanted Silver

The amount of silver incorporated in each sample is presented in Table 9.

**Table 9:** Measurement of Silver Content in Implanted Samples by X-Ray Fluorescence

sample	5 keV −3 V	5 keV −6 V	10 keV −3 V	10 keV −6 V
**silver amount** (**μg**/**cm** ^**2**^)	11.4 ± 0.7	17.5 ± 0.9	12.6 ± 1.9	19.3 ± 1.2
**silver amount**(**atoms**/**cm** ^**2**^)	6.3 ± 0.4 × 10^16^	9.8 ± 0.5 × 10^16^	7.0 ± 1 × 10^16^	1.1 ± 0.07 × 10^17^

The results indicate that samples implanted using the same thickness meter setting (3 or 6 V) exhibit very similar ion concentrations, as expected, since the implantation voltage in keV primarily determines the penetration depth of the ions rather than their total quantity. However, when the meter setting is increased from 3 to 6 V, an increase in the amount of silver incorporated into the samples is observed.

The quantity of silver implanted in this study is consistent with previous reports in the literature that exploit the properties of this biocompatible material. For instance, Zilio et al.^46^ demonstrated that silver implanted into stainless steel at a concentration of 3.5 × 10^16^ atoms/cm^2^, with a penetration depth of 5 nm, was capable of inhibiting the growth of S. Enteritidis and *L. monocytogenes*. Similarly, Ni et al.^47^ implanted Ag^+^ ions into stainless steel at a dose of 2 × 10^17^ atoms/cm^2^, achieving bactericidal activity against *E. coli*. Zimmerman et al.,^48^ in turn, implanted Ag^+^ into biocompatible glassy polymeric carbon (GPC) at a concentration of 5 × 10^16^ atoms/cm^2^. This dose was sufficient to enhance the material’s biocompatibility, rendering it potentially suitable for cardiac valve construction. The silver ions inhibited the adhesion of surrounding tissue cells, which is advantageous in applications where reduced risk of embolism is desired.

### Cell Viability Test

Failure of an orthopedic implant due to infection is an increasing concern. In many cases, bacterial contamination can progress to osteomyelitis, a condition affecting the bone or bone marrow. These complications typically occur at the surgical site or the site of device implantation. When the implant site is compromised, treatment is often challenging, and if antibiotics prove ineffective, revision surgery may be required. In light of the growing bacterial resistance to antibiotics, the use of medical devices incorporating silver ions has emerged as an effective strategy.^49^

However, silver ions can be cytotoxic to host cells depending on the concentration used, as they interact with various intracellular biomolecules, including nucleic acids, cell wall components, metabolic enzymes, and a wide range of proteins.^50^ Additionally, these ions can generate reactive oxygen species (ROS), resulting in genotoxicity and disruption of the cell cycle.^51^ Ionic release of silver is governed by the diffusion of fluids through the pores present on the coating surface. Although Ag^+^ ions exhibit antibacterial activity with relatively low toxicity, it is crucial to incorporate a minimal amount of silver to mitigate adverse effects on host tissues.^52^

The results of the indirect cell viability assay, presented in Figure 7, indicate that all samples containing Ag^+^ maintained cell viability within the standards established by ISO 10993-12. According to this standard, a material is considered cytotoxic when the reduction in cell viability exceeds 30% after 24 h of exposure. Nevertheless, samples with higher silver ion concentrations, characterized by a 6 V thickness meter setting, showed reduced viability. Literature suggests that the cytotoxicity of metallic ions in cell cultures is dose-dependent; the greater the number of ions incorporated into the sample, the higher the likelihood of cellular toxicity.^51^ The −5 keV −6 V sample showed results below 100% on the first day but recovered by the second day. In contrast, the −10 keV −6 V sample, which contained the highest silver concentration among all coatings produced (1.1 ± 0.07 × 10^17^ ions/cm^2^), exhibited viability below 100% on both days. This finding aligns with literature reports; for example, Fiedler et al. observed in indirect MTT assays that samples implanted with 1 × 10^16^ ions/cm^2^ maintained osteoblast viability, whereas doses of 1 × 10^17^ ions/cm^2^ reduced cell viability.^53^

**Figure 7:**
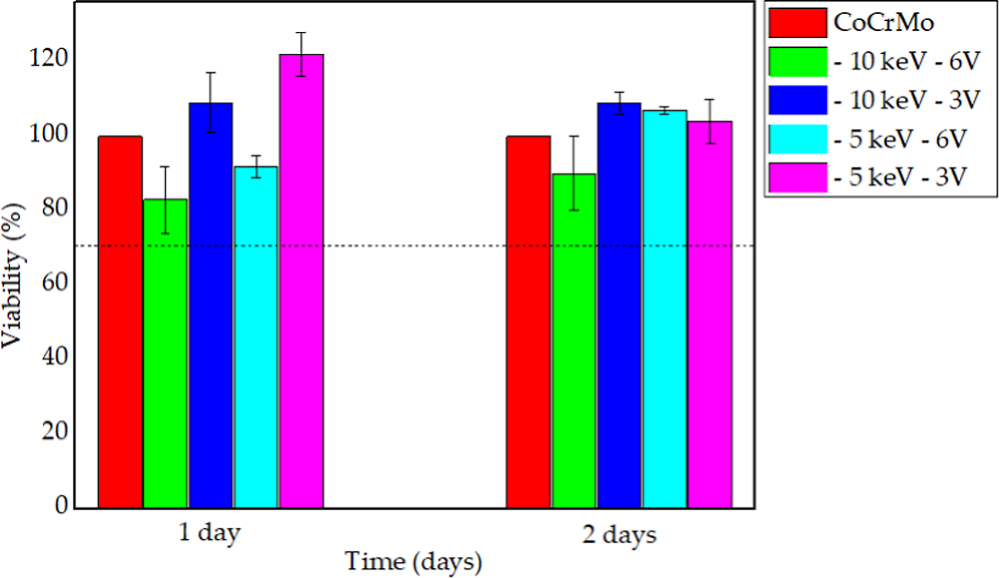
Results of the indirect MTT assay for the extracts compared to the control. The dashed line represents 70% viability, the minimum threshold required to demonstrate cell viability.

The uncoated CoCrMo sample may exhibit a certain degree of cytotoxicity under specific biological conditions. This behavior is associated with the release of metallic ions, particularly cobalt and chromium, which are well-recognized for their cytotoxicity to human cells.^54^ An increase in cell viability was observed in the coated samples on the second day compared to the control, which may be attributed to the presence of niobium-based coatings. These films act as protective barriers, significantly reducing the release of heavy metallic ions from the substrate, thereby improving the biocompatibility of the material.

To contextualize our findings, recent studies have similarly emphasized the need to balance antimicrobial performance with cytocompatibility in biomedical coatings. For instance, Akinay et al.^55^ demonstrated that POSS-modified Ti_3_C_2_T_*x*_ MXene films provide strong antibacterial inhibition against *E. coli* and *S. aureus* while maintaining high fibroblast cell viability at moderate concentrations, thereby illustrating how controlled surface chemistry can sustain antimicrobial effectiveness without inducing excessive cytotoxicity. This aligns with current approaches in Ag-based systems, where surface-confined silver configuration is preferred to ensure effective bacterial suppression with reduced Ag^+^ exposure to mammalian cells. Together, these findings reinforce the relevance of strategies that optimize antibacterial–cytocompatibility balance in next-generation biomedical coatings.

### Evaluation of Antibacterial Activity

In this test, the samples were exposed to two different bacterial strains: *S. aureus* and *E. coli*. Slight inhibition halos were observed in three samples (Figure 8). These samples include one exposed to *S. aureus* (Nb_2_N + Ag – 5 keV −6 V) and two exposed to *E. coli* (Nb_2_N + Ag – 10 keV −3 V and Nb_2_N + Ag – 5 keV −3 V). Although silver ions are widely recognized for their ability to kill both Gram-positive bacteria, such as *S. aureus*, and Gram-negative bacteria, such as *E. coli*,^56^ Gram-positive bacteria are generally more difficult to eradicate due to their thick cell wall (20–80 nm), composed primarily of peptidoglycan. In contrast, Gram-negative bacteria have a thinner peptidoglycan layer (<10 nm).^57^

**Figure 8:**
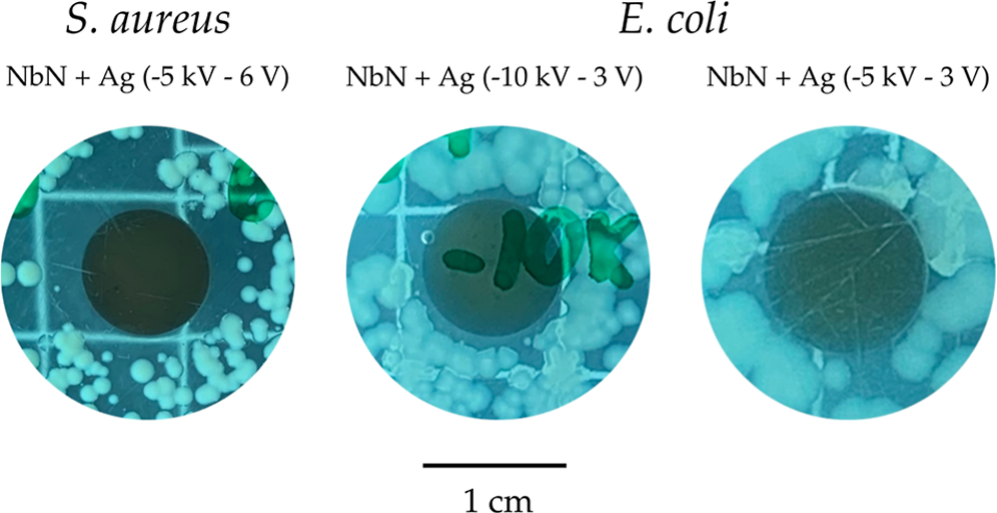
Inhibition halo formation in samples exposed to Gram-positive and Gram-negative bacteria.

The mechanisms underlying the antimicrobial activity of silver ions involve their affinity for sulfur, oxygen, and nitrogen atoms present in bacterial cells, leading to the formation of silver salts and disruption of essential biochemical processes. Additionally, silver ions interact with thiol and amino groups in proteins, producing deleterious effects.^49^

Furthermore, it was observed that samples containing silver incorporated into Nb_2_N films exhibited more pronounced bactericidal activity compared to those incorporated into Nb_2_O_5_ films. As demonstrated in the Monte Carlo simulation section, the denser niobium nitride film causes greater energy loss of the ions upon penetration, resulting in their deposition in interstices near the surface. In contrast, the less dense niobium oxide film allows deeper ion penetration. In the context of implants, once inserted into the human body, the inert surface is rapidly coated with tissue proteins such as fibrinogen, fibronectin, and collagen, which serve as adhesive substrates for microbial attachment.^58^ Therefore, a rapid release of silver ions is required, which is facilitated by denser coatings such as Nb_2_N, while still allowing for sustained ion leaching to prevent future contamination.

In addition to the three samples exhibiting bactericidal activity mentioned above, Figure 9 shows that the remaining samples with silver-implanted CoCrMo substrates exhibited bacteriostatic activity. That is, they were unable to kill bacteria but inhibited biofilm formation in the regions of contact with the agar. This was evident upon removal of the samples from the Petri dishes, as no bacterial colonies appeared on the silver-containing samples, whereas growth was observed on uncoated CoCrMo and CoCrMo with Nb_2_N and Nb_2_O_5_ films, which are neither bactericidal nor bacteriostatic. Figure 9 also includes images of the samples under DAPI staining and ultraviolet light. No fluorescent spots indicative of bacterial DNA were observed in the silver-containing samples, confirming the absence of bacterial presence.

**Figure 9:**
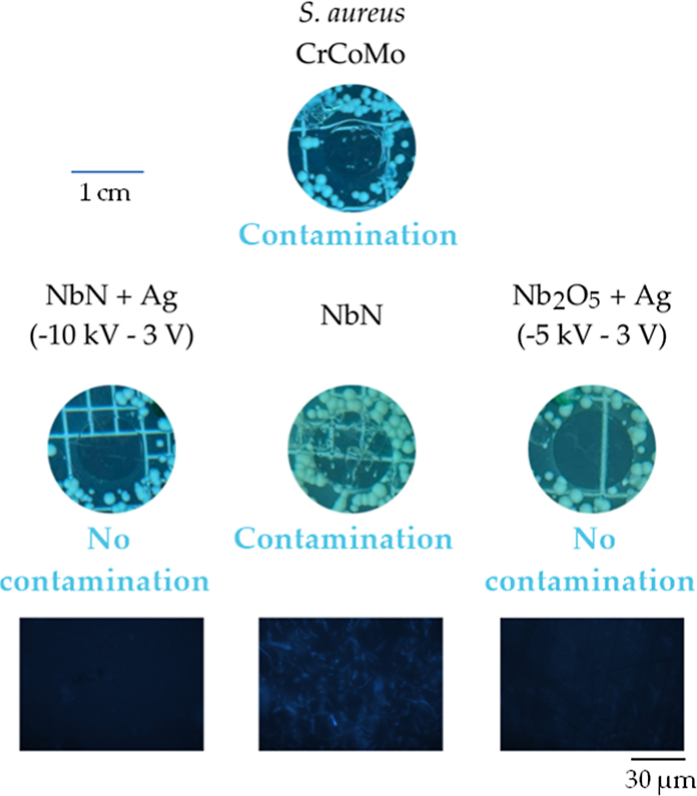
Representation of bacteriostatic activity in CoCrMo samples implanted with silver ions compared to uncoated or niobium-coated samples without silver.

The first 24 h after implantation are the most important for determining the antibacterial performance of modified surfaces, as this period requires an early release of antimicrobial species, followed by a slower and continuous lixiviation over longer periods. In the present work, this behavior is favored by the low-energy ion plating technique employed, which differs from conventional ion implantation primarily in the implantation energy. While traditional processes operate at high energies (≥500 keV), driving implanted ions deeper into the material and delaying their release, the approach used here operates at lower energies (≤10 keV), retaining antibacterial ions closer to the surface and enabling their availability within the first 24 h. Previous studies from our research group support this mechanism, demonstrating reduced microbial adhesion on samples containing ions implanted at low density after 24 h of contact, attributed to the near-surface distribution of active species.^44,46^ Therefore, the assessment of antibacterial activity under direct-contact conditions becomes particularly relevant, as it better mimics the physiological scenario in which long-distance diffusion is limited.

### Evaluation of Wettability and Surface Roughness

In order to further reduce the sample set for this study, the samples exhibiting the best bactericidal performance were selected for further investigation. Additionally, samples implanted with silver at 10 keV were not chosen, as this high voltage was causing electrical issues in the ion plating equipment.

The contact angle measurements obtained indicate that the niobium-based thin films with incorporated silver maintain the hydrophilic character of the CoCrMo substrates (Table 10). Furthermore, the values for Nb_2_N and Nb_2_O_5_ films were similar, as expected based on literature reports. For instance, Ramírez et al.^24^ deposited both coatings on stainless steel and measured contact angles of 78.7° for NbN and 80.7° for Nb_2_O_5_. In the context of the present study, where the films are intended for application in dental prostheses, the results suggest that the biocompatibility of the metallic substrate was improved.

**Table 10:** Contact Angle Results for the Samples

sample	contact angle (°)
CoCrMo	72.3 ± 2.8
CoCrMo + Nb_2_N	66.7 ± 2.3
CoCrMo + Nb_2_N + Ag (5 keV −6 V)	60.8 ± 1.1
CoCrMo + Nb_2_N + Ag (5 keV −3 V)	38.6 ± 5.6
CoCrMo + Nb_2_O_5_	66.6 ± 3.2
CoCrMo + Nb_2_O_5_ + Ag (5 keV −6 V)	67.6 ± 4.7

These findings reinforce the evidence that hydrophilic surfaces—i.e., those with a contact angle below 90°—are advantageous for biomedical applications. The literature emphasizes the importance of hydrophilic surfaces in promoting the adsorption of plasma proteins, which are essential for early osteogenic interactions.^59^ Moreover, hydrophilicity provides substantial benefits during the initial stages of healing and throughout the osseointegration process, facilitating bone integration. The positive impact of hydrophilicity on osseointegration is further reflected in improvements in bone-implant contact (BIC) and bone anchorage during the early consolidation phases of the implant.^60^

Future studies should include monitoring the contact angle over time to assess the shelf life of the coatings and verify the maintenance of their hydrophilic character.

Table 11 presents the roughness values obtained for the samples. Surface roughness is a critical parameter in the performance of implantable biomaterials, as it directly influences biological phenomena such as cell adhesion, proliferation, and spreading, in addition to bacterial biofilm formation.^61^ According to the literature, surfaces with an average roughness (Ra) below 0.4 μm, as observed for samples with CoCrMo substrates, are classified as smooth. Scientific evidence indicates that smooth surfaces tend to hinder bacterial adhesion, thereby reducing the risk of microbial colonization and infection around the implant. This occurs because many bacterial species preferentially attach to microtopographically rougher surfaces, which provide greater anchorage area and protection against shear forces.^61^

**Table 11:** Surface Roughness Results for the Samples

Sample	roughness Ra (μm)
CoCrMo	0.024 ± 0.001
CoCrMo + Nb_2_N	0.026 ± 0.001
CoCrMo + Nb_2_N + Ag (5 keV −6 V)	0.046 ± 0.002
CoCrMo + Nb_2_N + Ag (5 keV −3 V)	0.041 ± 0.001
CoCrMo + Nb_2_O_5_	0.033 ± 0.002
CoCrMo + Nb_2_O_5_ + Ag (5 keV −6 V)	0.054 ± 0.004

### Quantitative Corrosion Testing

As presented in Figure 10 (OCP test without friction), the uncoated CoCrMo sample exhibited an average potential of approximately +0.023 V, a behavior similar to that observed for the Nb_2_N film containing implanted silver (Nb_2_N + Ag). Both samples demonstrated the best anticorrosive performance, indicating the maintenance of a stable and passive surface environment. The excellent corrosion resistance of the CoCrMo alloy is related to the spontaneous formation of a chromium oxide (Cr_2_O_3_) passive layer, which acts as a protective barrier against aggressive ions.^62^

**Figure 10:**
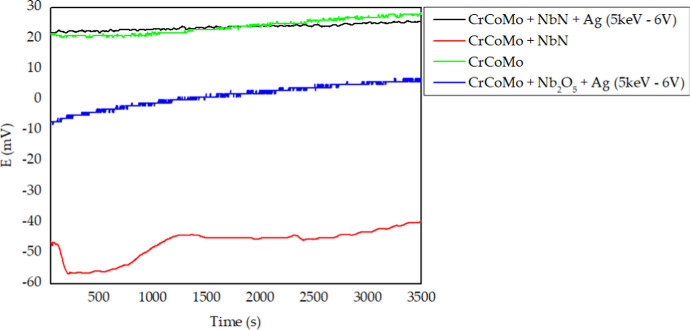
Results of the OCP test without friction.

In the case of the Nb_2_N ceramic film containing implanted silver, the favorable performance can be attributed to the high hardness and density of the nitride, which significantly reduces ion permeability, coupled with the effect of silver ion implantation, which may seal structural microdefects and densify the surface.^63,64^ Furthermore, silver possesses recognized antioxidant activity, which can inhibit undesirable redox reactions at the film–substrate interface.^65^

Conversely, the Nb_2_N-only film exhibited a more negative potential relative to pure CoCrMo, suggesting a reduced corrosion resistance. This behavior may be associated with possible structural defects in the film, such as pores, microcracks, or adhesion failures, which act as preferential pathways for the penetration of corrosive species. Such features compromise the integrity of the protective barrier, promoting localized pitting corrosion.^66^

Silver ion implantation appears to significantly mitigate these defects, as observed in the Nb_2_O_5_ + Ag (5 keV −6 V) coating, where implanted ions can occupy interstitial sites within the crystal structure and improve film compaction, rendering it less permeable to ions and more resistant to corrosive reactions.^67^ This surface modification also reinforces the antioxidant role of silver reported in the literature,^65^ representing a promising approach for both corrosion protection and antimicrobial applications. Additionally, as discussed in previous sections, harder films tend to be more effective at preventing corrosive processes by limiting the diffusion of harmful ions from the environment into the substrate.^39^ Literature reports indicate that Nb_2_N coatings possess higher hardness than Nb_2_O_5_ coatings, which may explain the superior performance of the former over the latter.^68^

Mass measurements before and after the tribocorrosion tests provide deeper insight into sample behavior and are presented in Table 12. For example, the uncoated CoCrMo sample exhibited one of the best electrochemical potentials in the OCP tests under static conditions. However, after the tribocorrosion test, this sample showed the highest mass loss among all evaluated samples (0.002 g). This result demonstrates that OCP data under static conditions may not adequately reflect material susceptibility in dynamic environments, such as tribocorrosion tests or in vivo applications.

**Table 12:** Mass Variation of Samples Before and After Tribocorrosion Tests

sample	mass before the tribocorrosion test (g)	mass after the tribocorrosion test (g)
**CoCrMo**	0.356	0.354
**CoCrMo** + **Nb**_**2**_ **N** + **Ag** (**5 keV** – **6 V**)	0.358	0.357
**CoCrMo** + **Nb**_**2**_ **O**_**5**_ + **Ag** (**5 keV** – **6 V**)	0.374	0.374
**CoCrMo** + **Nb**_**2**_ **N**	0.370	0.370

The spontaneous formation of the Cr_2_O_3_ passive layer on the CoCrMo surface provides effective protection in physiological media (FBS) as long as the surface remains intact. However, continuous removal of this protective film by mechanical friction successively exposes the substrate to the corrosive environment, favoring localized degradation processes, such as pitting corrosion.

Conversely, the CoCrMo sample coated with a Nb_2_N ceramic film and subjected to silver implantation exhibited a mass loss of only 0.001 g, lower than that of the uncoated sample. Despite having a similar OCP potential under static conditions (both around 0.023 V), its superior tribocorrosion performance demonstrates that corrosion protection is more effective under frictional conditions. This efficiency is associated with both the high hardness of the Nb_2_N film and the action of the implanted silver, which may fill microdefects and provide localized antioxidant effects. Furthermore, as previously observed, the wear coefficient for this sample (0.37 × 10^–12^ m^2^/N) was significantly lower than that of pure CoCrMo (0.91 × 10^–12^ m^2^/N), reinforcing the mechanical resistance of the system under tribological action.

Even more notably, samples coated solely with Nb_2_N thin films exhibited no significant material loss during the test. Interestingly, the CoCrMo sample with this coating showed the most negative OCP potential among all tested (≈− 0.060 V), which would initially suggest higher oxidation propensity. However, tribocorrosion performance contradicts this expectation, revealing the limitations of interpreting static OCP data in isolation. Thus, even with a more negative potential, the presence of a stable low-friction coating may have been decisive in limiting corrosion progression during wear.

These results clearly demonstrate that tribocorrosion resistance does not depend solely on electrochemical characteristics measured under open-circuit conditions. System performance is strongly conditioned by coating integrity, adhesion, and wear coefficient. Dense, homogeneous ceramic films exhibit superior behavior in aggressive environments where wear and corrosion occur simultaneously.

As illustrated in the tribocorrosion graphs in Figure 11, the uncoated CoCrMo sample showed a gradual increase in corrosion potential (E), starting at approximately 29 mV and reaching around 44 mV after 7200 s. This behavior indicates the progressive formation of a surface passive layer composed mainly of chromium oxides (Cr_2_O_3_),^69^ typical for this alloy in physiological media such as FBS. Formation of this passive layer helps fill surface irregularities, resulting in a thin, continuous film that reduces friction between contacting surfaces, reflected in lower coefficients of friction (COF). However, over time, continuous mechanical wear can gradually remove this passive layer, leading to increased COF and decreased corrosion potential due to substrate exposure.^70^

**Figure 11:**
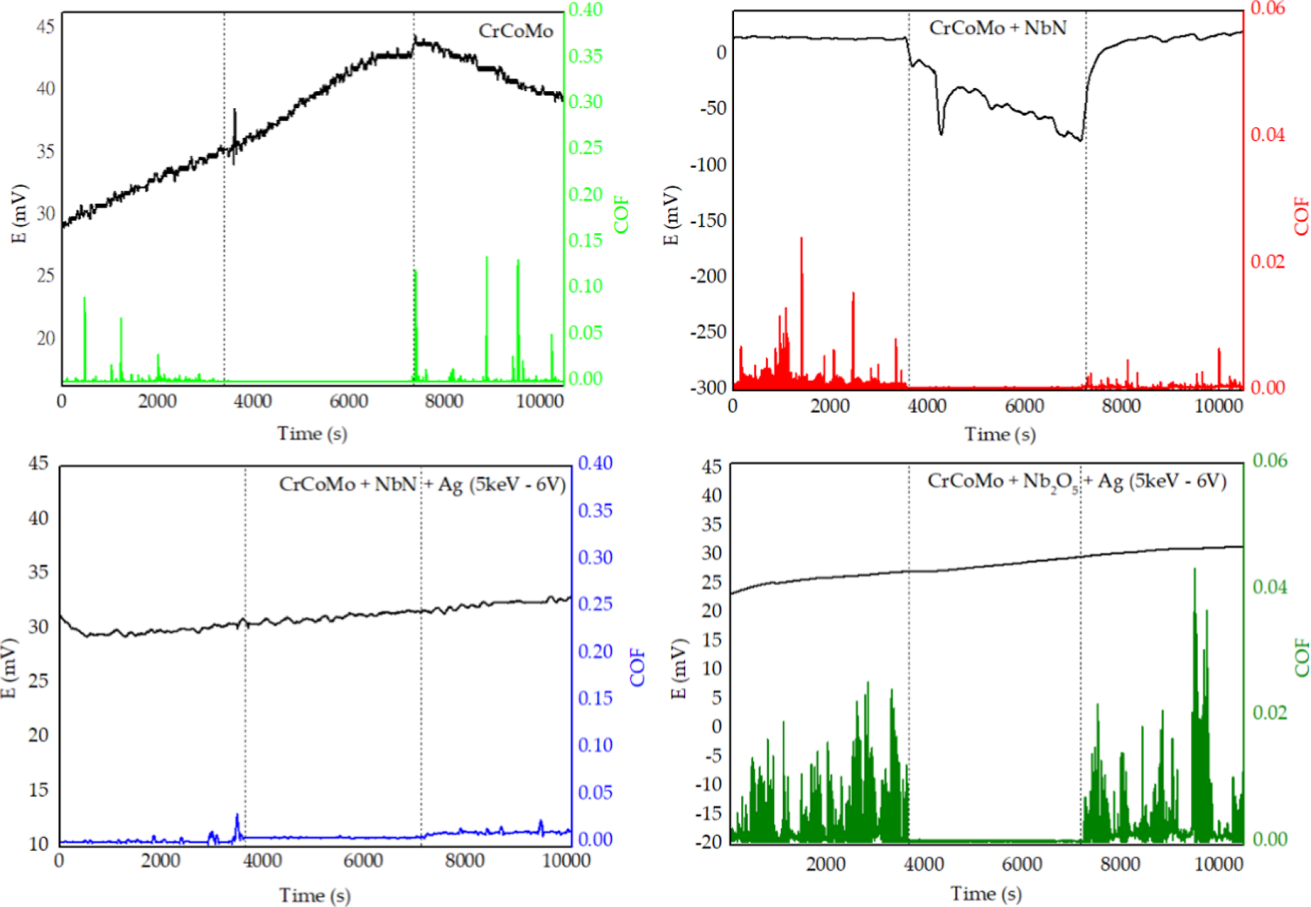
Tribocorrosion results for samples with CoCrMo substrate.

The CoCrMo sample coated with Nb_2_N exhibited initially stable electrochemical behavior, indicating the film’s effectiveness as a protective barrier. However, around 3600 s, an abrupt drop in corrosion potential was observed, reaching negative values. This sudden change suggests localized rupture of the Nb_2_N film in the tribological contact zone, exposing the substrate and activating corrosive processes.^71^ Simultaneously, a significant reduction in COF was observed, likely due to the formation of an intermediate tribochemical layer composed of corrosion products, such as cobalt or molybdenum hydroxides, acting as temporary lubricants until the system reaches new stability.^72^ After this critical period, corrosion potential gradually recovered, indicating partial repassivation. This demonstrates that while the Nb_2_N film provides initial protection against corrosion and wear, its durability under tribocorrosion is limited. Partial potential recovery suggests that failure is not catastrophic and passive regeneration occurs. For long-term applications in aggressive environments such as the human body, additional modifications, such as ion implantation, are necessary to ensure long-term stability.

The CoCrMo sample coated with Nb_2_N and implanted with silver exhibited a stable corrosion potential throughout the test, with a slight upward trend, indicating high electrochemical stability and no surface passivation rupture. Notably, COF remained practically constant over time. This tribological stability suggests favorable surface contact, with significantly reduced mechanical interaction. The presence of silver in the NbN film is a key factor in this performance, as silver may fill microdefects and provide local antioxidant effects.^73^

The Nb_2_O_5_ + Ag coating also showed positive evolution over time, indicating electrochemical surface stability. This trend suggests that the coating presents protective characteristics that inhibit corrosion progression even after prolonged exposure to an aggressive medium. The COF remained low, indicating satisfactory tribological behavior, likely due to the presence of silver and the ceramic nature of Nb_2_O_5_, which contributes to wear resistance.

To reconcile these findings, it is important to distinguish between static passivation response and dynamic wear-induced degradation. OCP measurements provide information on the stability of surface films in the absence of mechanical interaction and are therefore governed primarily by passivation kinetics and defect sensitivity.^74^ In contrast, tribocorrosion performance depends not only on electrochemical nobility but also on the ability of a coating to withstand mechanical removal of passive layers, maintain surface integrity, and minimize frictional wear. As demonstrated in our results, samples with favorable OCP values (such as uncoated CoCrMo) may still experience mass loss under tribocorrosive conditions, while coatings with more negative OCP values (such as Nb_2_N) can exhibit minimal material loss due to their dense ceramic nature and low-friction behavior. Therefore, static OCP data and dynamic tribocorrosion performance should be interpreted as complementary rather than contradictory, reflecting distinct degradation regimes that coexist in biomedical applications.

Also, it is well established that serum proteins can adsorb onto biomaterial surfaces and influence both passive film stability and corrosion behavior in physiological environments. For example, Karimi et al. reported that bovine serum albumin (BSA) interacts with the passive oxide film on CoCrMo and other biomedical alloys, affecting electrochemical characteristics and corrosion rates, with higher BSA concentrations enhancing passive film stability in some cases.^75^ Similarly, Taufiqurrakhman et al.^76^ have shown that protein presence can modify surface properties during tribocorrosion by altering the exposed interface once passive layers are disrupted, suppressing the dissolution of metal ions even before the reformation of the passive film. In the context of our results, the simulated physiological environment containing FBS likely promotes adsorption of serum proteins onto the CoCrMo and coating surfaces, which could contribute to the observed corrosion behavior by modifying passive film formation, compaction, and stability under static and dynamic conditions. Integrating these literature findings supports a more comprehensive interpretation of our corrosion and tribocorrosion data and the complex role of protein–surface interactions.

#### X-ray Photoelectron Spectroscopy (XPS)

Through X-ray photoelectron spectroscopy (XPS) analysis, it was possible to evaluate the chemical bonding states of the elements present in the samples that exhibited the best performance in biological assays. The CoCrMo samples coated with niobium nitride films and implanted silver — 5 keV −6 V (Figure 12) and 5 keV −3 V (Figure 13) — were analyzed, revealing the surface presence of Ag, N, Nb, O, and C.

**Figure 12:**
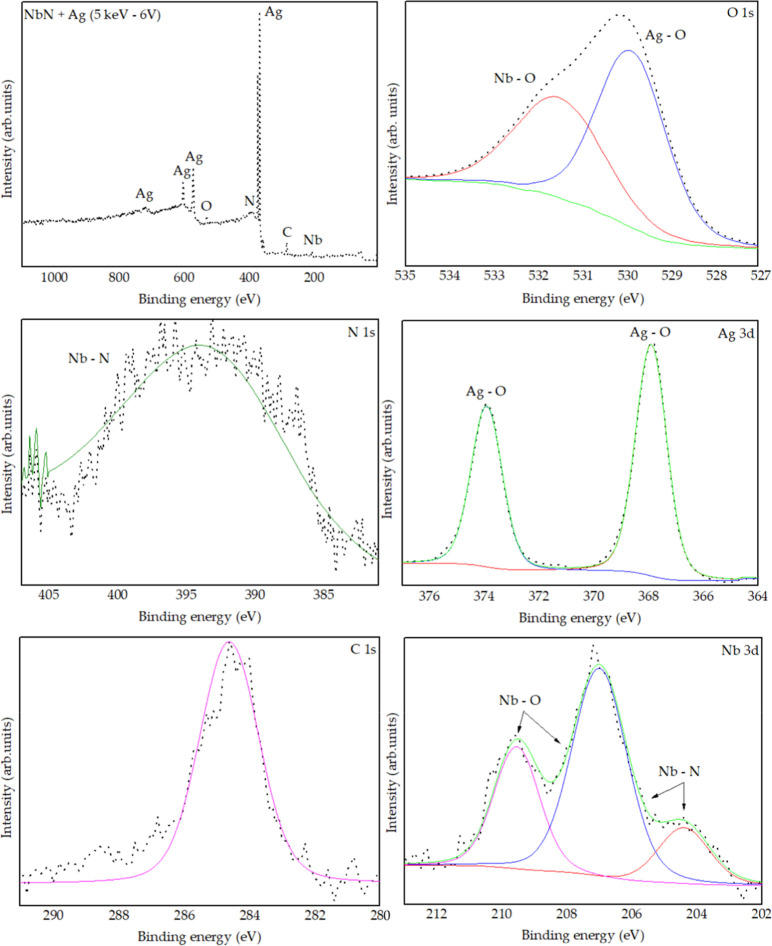
XPS analysis of the NbN + Ag (5 keV −6 V) sample.

**Figure 13:**
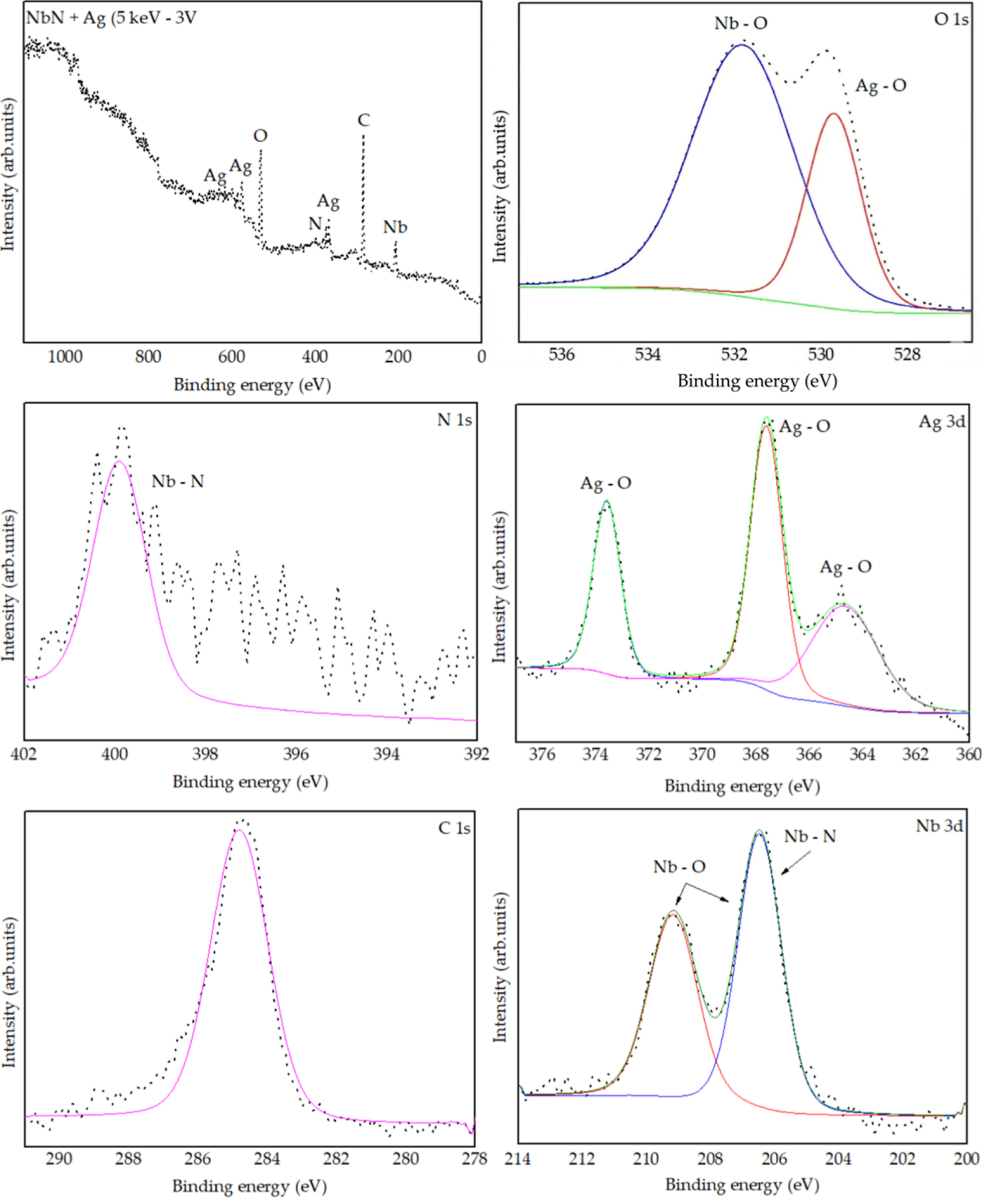
XPS analysis of the NbN + Ag (5 keV −3 V) sample.

The region between 204 and 210 eV, corresponding to the Nb 3d transition, exhibited the following peaks: one at ∼204 eV, associated with the Nb–N bond (Nb 3d_5_/_2_) [131]; another centered at ∼207 eV, attributed to an overlap of Nb–N and Nb–O states (Nb 3d_5_/_2_ and Nb 3d_3_/_2_);^77,78^ and a third near 210 eV, related to Nb–O bonding (Nb 3d_3_/_2_). In the range of 384–404 eV, a peak at ∼397 eV was observed, corresponding to the N 1s state of the Nb–N bond.^79^ The silver–oxygen bond (O 1s) was identified at ∼529 eV, indicating the presence of Ag_2_O,^80^ and the niobium–oxygen bond was identified at ∼531 eV, indicating the presence of Nb_2_O_5_. A peak at 284.6 eV was assigned to the C 1s state, typical of surface organic contaminants. Silver also presented two characteristic peaks at 367.9 eV (Ag 3d_5_/_2_) and 373.9 eV (Ag 3d_3_/_2_), with a 6 eV separation — a clear indicator of Ag_2_O formation, as reported in the literature.^81,82^

Comparison between the 5 keV −6 V and 5 keV −3 V samples revealed subtle spectral differences. The 5 keV −3 V sample exhibits an additional satellite peak at ∼365 eV associated with silver. These secondary peaks, often located to the right of the main peaks, reflect additional electronic effects. In compounds such as Ag_2_O, these satellites can be related to complex electronic structures and shakeup transitions, in which a valence electron is excited simultaneously with the ejection of the primary photoelectron. Additionally, this sample does not show the peak at ∼204 eV, suggesting lower chemical complexity and a reduced presence of mixed niobium states compared to the 5 keV −6 V sample.

The results indicate that both the niobium nitride film and the implanted silver underwent oxidation. This oxidation may result from various factors, including insufficient physical cleaning (etching), residual oxygen in the vacuum chamber, or natural surface oxidation. Nevertheless, the literature demonstrates that silver retains its bactericidal activity even when oxidized, as the release of Ag^+^ ions — the main antimicrobial agent — is not compromised and may even be enhanced. Manikandan et al.^83^ demonstrated that Ag_2_O nanoparticles exhibit strong antibacterial activity against *Streptococcus mutans* and Lactobacilli spp. Similarly, Bellantone et al.^84^ showed that bioactive glasses containing Ag_2_O effectively inhibit the growth of *E. coli*, *Pseudomonas aeruginosa*, and *S. aureus*, with the effect attributed to the leaching of Ag^+^ ions from the matrix.

The apparent oxidation of niobium nitride observed in the XPS spectra, but not in the XRD diffractograms (which only identified Nb_2_N peaks), can be explained by the difference in analysis depth between the techniques. While XPS probes the surface layer (∼10 nm), XRD analyzes significantly larger volumes (tens to hundreds of nanometers). Therefore, it is likely that only the surface of the film is oxidized. Furthermore, the oxide layer may be amorphous, rendering it undetectable by XRD.

Is is important to highlight that the XPS spectrum of the O 1s region was fitted with two distinct components to resolve the different oxygen chemical environments present on the surface. The higher binding energy component, centered at approximately ∼531 eV, is assigned to lattice oxygen (O^2–^) associated with metal–oxygen bonds, consistent with typical O 1s binding energies of metal oxides reported in the literature (e.g., Nb_2_O_5_).^85,86^ The lower binding energy component, observed at ∼529.0 eV, is attributed to oxygen species, such as oxygen atoms bound in different chemical environments including surface hydroxyls, adsorbed oxygen, or oxidized silver–oxygen bonds.^87^ In studies of mixed metal oxides, two-component O 1s fits are routinely used to distinguish lattice O^2–^ from lower-energy oxygen species related to surface defects or adsorbates, highlighting the importance of peak deconvolution for accurate chemical state analysis in XPS.^86^ In our case, the lower binding energy O 1s component correlates well with the presence of oxidized silver (Ag–O) species detected in the Ag 3d region, supporting the conclusion that the surface contains both lattice oxygen associated with oxide formation and additional oxygen species linked to Ag-oxidation.

### Correlations between the Physicochemical, Tribological, and Biological Properties of the Developed Coatings

The integration of the results obtained throughout this study highlights a strong interdependence between the physicochemical, tribological, and biological properties of thin films of NbN and Nb_2_O_5_, both pure and incorporated with silver. The observed correlations provide an in-depth understanding of the coatings’ behavior in biomedical applications, emphasizing the importance of controlling variables such as thickness, stoichiometry, morphology, and chemical composition.

Increasing the film thickness, achieved through longer deposition times or higher power, promoted the formation of larger grains and a reduced density of grain boundaries, which are often critical regions for the initiation of structural failures. This more homogeneous microstructure is directly associated with lower susceptibility to corrosion and wear, as evidenced by the lower wear coefficients observed in thicker samples, such as Nb_2_O_5_ deposited at 90 W and Nb_2_N deposited for 1 h. The latter also exhibited the β-Nb_2_N phase, structurally denser and more resistant than the cubic δ-NbN phase.

The crystalline structure was also found to be a decisive factor for film performance. The predominantly amorphous character contributed to enhanced corrosion resistance, due to the absence of defects typical of crystalline materials. Moreover, the higher density of Nb_2_N films restricted silver ion penetration, concentrating them near the surface—a condition ideal for immediate bactericidal effects. In contrast, Nb_2_O_5_ allowed deeper ion penetration, favoring a prolonged, although less intense, antimicrobial effect. These features explain the differential performance observed against microorganisms.

Film biocompatibility was directly related to composition and surface morphology. The coatings reduced the cytotoxicity associated with the CoCrMo substrate by acting as barriers to the release of heavy metal ions. The hydrophilic surface, combined with controlled roughness, promoted cell integration while simultaneously reducing bacterial adhesion.

From an electrochemical standpoint, silver ion implantation was crucial for improving corrosion resistance. By filling microdefects and densifying the surface, the silver ions increased the open-circuit potential stability and reduced mass loss during tribocorrosion tests, even under dynamic wear conditions.

The correlation of data obtained through multiple characterization techniques reinforces that the overall system performance does not rely on a single property, but on the synergy between them. The suitability of the films for the proposed applications results from the combination of mechanical strength, chemical stability, favorable biological response, and antimicrobial behavior. This integrated surface-engineering approach demonstrates the potential of NbN and Nb_2_O_5_ coatings with implanted silver as multifunctional solutions capable of simultaneously meeting the technical and biological requirements imposed by critical environments.

## Conclusions

This work aimed to develop and characterize niobium-based coatings incorporated with silver, suitable for biomedical applications. NbN and Nb_2_O_5_ coatings were deposited via magnetron sputtering and subsequently modified through silver ion implantation using the ion plating technique. The results obtained throughout the study demonstrated that these films exhibit promising physicochemical and biological properties for the intended applications.

In the biomedical context, the coatings proved effective in terms of biocompatibility, wear resistance, and antibacterial behavior—critical aspects for the safety and longevity of orthopedic implants. Hydrophilic surface formation, which hinders bacterial adhesion, and the absence of significant cytotoxicity toward bone cells were particularly noteworthy, confirming the suitability of the films for contact with biological tissues. Based on the tests performed, the CoCrMo + Nb_2_N + Ag (5 keV −6 V) sample is recommended for biomedical applications, as it demonstrated the most satisfactory behavior regarding cellular response, antibacterial activity, wear, and corrosion resistance.

The combination of deposition and implantation techniques, along with careful selection of processing parameters, resulted in stable and uniform coatings. Both physicochemical and biological assessments validated the central hypothesis of the study: surface engineering can serve as an effective and eco-friendly strategy to combat corrosion, wear, and microbial proliferation in critical sectors of healthcare.

For future perspectives, further in vitro studies are recommended, along with the evaluation of long-term durability and performance under extreme operating conditions. The scalability of the technique should also be explored, aiming at industrial application of the developed coatings.

Beyond the scientific findings, the developed coating system presents clear clinical relevance and translational potential. Orthopedic implants remain highly susceptible to infection, wear, and corrosion, leading causes of implant failure and revision surgeries. By improving antibacterial performance, mitigating surface degradation, and maintaining biocompatibility, the proposed Nb-based coatings align with unmet medical needs in orthopedics and trauma care. Furthermore, the use of established physical vapor deposition and ion implantation technologies facilitates potential integration into existing manufacturing workflows, supporting regulatory and industrial translation. As such, the developed system represents a promising pathway toward next-generation implant surfaces designed to enhance patient outcomes and reduce healthcare burdens.
